# LPIN1 Is a Regulatory Factor Associated With Immune Response and Inflammation in Sepsis

**DOI:** 10.3389/fimmu.2022.820164

**Published:** 2022-02-09

**Authors:** Wei Dai, Ping Zheng, Deqiang Luo, Qian Xie, Fen Liu, Qiang Shao, Ning Zhao, Kejian Qian

**Affiliations:** ^1^ Department of Intensive Care Unit, The First Affiliated Hospital of Nanchang University, Nanchang, China; ^2^ Medical Innovation Center, The First Affiliated Hospital of Nanchang University, Nanchang, China; ^3^ Department of Intensive Care Unit, The Fifth Dongxin’s Hospital of Shangrao City, Shangrao, China; ^4^ Department of Key Laboratory, Shanghai Pudong New Area People’s Hospital, Shanghai, China

**Keywords:** LPIN1, sepsis, immune infiltration, inflammation, NF-κB

## Abstract

**Objectives:**

Sepsis is a clinical disease that is typically treated in the intensive care unit, and the complex pathophysiology under this disease has not been thoroughly understood. While ferroptosis is involved in inflammation and infection, its effect in sepsis is still unknown. The study aimed to identify ferroptosis-related genes in sepsis, providing translational potential therapeutic targets.

**Methods:**

The dataset GSE65682 was used to download the sample source from the Gene Expression Omnibus (GEO) database. Consensus weighted gene co-expression network analysis (WGCNA) was performed to find suspected modules of sepsis. The differentially expressed genes (DEGs) most significantly associated with mortality were intersected with those altered by lipopolysaccharide (LPS) treatment and were further analyzed for the identification of main pathways of Gene Ontology (GO) and Kyoto Encyclopedia of Genes and Genomes (KEGG) analysis. The related pathway markers were further verified by qPCR.

**Results:**

A total of 802 blood samples with sepsis were included for WGCNA, which identified 21 modules. Intersected with ferroptosis databases and LPS treatment groups, we identified two ferroptosis-related genes: PEBP1 and LPIN1. Only LPIN1 contributes to a poor outcome. Then, 205 DEGs were further identified according to the high or low LPIN1 expression. Among them, we constructed a gene regulatory network with several transcriptional factors using the NetworkAnalyst online tool and identified that these genes mostly correlate with inflammation and immune response. The immune infiltration analysis showed that lower expression of LPIN1 was related to macrophage infiltration and could be an independent predictor factor of the survival status in sepsis patients. Meanwhile, the multivariate Cox analysis showed that LPIN1 had a significant correlation with survival that was further verified by *in vitro* and *in vivo* experiments.

**Conclusion:**

In conclusion, LPIN1 could become a reliable biomarker for patient survival in sepsis, which is associated with immune and inflammation status.

## Introduction

Sepsis is a clinical disease involved in multiple organ dysfunction caused by critical inflammation and infection (1). It can result in a higher mortality in critically ill patients (2), which accounts for one-third of hospitalized patients (3). Although sepsis has a high mortality and morbidity, few medicines have been proven to cure it. This might be due to the complex pathophysiology under this disease that has not been thoroughly understood.

Some studies have been performed regarding the genomic transcriptional analysis to explore the potential signaling pathways in sepsis (4–6). Scicluna et al. (5) reported the endotypes of sepsis based on a machine learning model and proposed that identification of sepsis endotypes was useful in personalized medicine management and selection for clinical trials. Antcliffe et al. (6) identified the sepsis response signatures (SRSs) according to transcriptomic data. Most of these studies focused on the biological pathways such as ferroptosis or genetic profiling; however, no exact gene has been datamined for this critical disease.

Ferroptosis was firstly identified by Dolma et al. (7) in cancer cells and was found to be different from apoptosis, pyroptosis, and necroptosis, which are also involved in neurotoxicity, kidney injury, liver injury, and heart disease (8–10). Due to its role in multiple organs, ferroptosis has also been implicated in sepsis (11). However, the genetic background of ferroptosis in sepsis remains elusive. Therefore, we applied a weighted gene co-expression network analysis (WGCNA) to link the potential differentially expressed genes (DEGs) in ferroptosis (12). To the best of our knowledge, WGCNA has been used to explore co-expression patterns in pediatric sepsis (13, 14), HIV infection (15), and *in vitro* inflammatory cells (16).

As sepsis might be a heterogeneous disease status and involve a series of onset genes, the present study aimed to identify gene co-expression modules in sepsis by WGCNA and to further link the modules to immune status and clinical features based on *in vitro* and *in vivo* experiments. The proposed hypothesis is that ferroptosis-related genes are implicated in sepsis and correlated with survival status.

## Materials and Methods

### Data Download and Data Preprocessing

We used bioinformatics and experimental methods to explore the biological characteristics of sepsis. First, we used the GEOquery package of the R software (version 4.1.0, http://r-project.org/) to download the sample source from the Gene Expression Omnibus (GEO) (https://www.ncbi.nlm.nih.gov/geo/) database. The reliable sepsis expression profile GSE65682 are all from *Homo sapiens*. GSE65682 is based on GPL13667[HG-U219] Affymetrix Human Genome U219 Array platform. This data set contains 802 blood samples, including 760 sepsis and 42 healthy controls. After being standardized, annotated, and cleared, the clinical information of data set GSE65682 was used for bioinformatic analysis. In the genecards database (https://www.genecards.org/), 304 ferroptosis-related genes were searched using ferroptosis as a keyword. A total of 288 ferroptosis-related genes as Driver, Suppressor, and Marker genes were also obtained from the FerrDb database (http://www.zhounan.org/ferrdb/). A total of 470 ferroptosis-related genes were obtained from the intersection of these two parts.

### RNA Sequencing and Data Analysis

Rat macrophage NR8383 cells were inoculated into a 6-well plate containing 2 ml medium per well at a density of 10^6^ cells. When the cells grew to 50%, the cells were stimulated with 1 μg/ml lipopolysaccharide (LPS) and collected after 2 h or 9 h, respectively. Extracted total RNA from LPS-treated cells or untreated NR8383 cells were submitted to The Beijing Genomics Institute (BGI) to construct a sequencing library, and RNA high-throughput sequencing was performed through the Illumina HiSeqTM2000 platform.

### Weighted Gene Co-Expression Network Analysis

For WGCNA, abnormal samples were deleted to ensure that the results of the network construction were reliable. First, the soft threshold for network construction was selected. The adjacency matrix is a continuous value between 0 and 1, so the constructed network conforms to the power-law distribution and is closer to the real biological state. Second, the block module function was applied to construct a scale-free network, and module division analysis was performed to identify gene co-expression modules. These modules are defined by gene branches using a dynamic tree cutting algorithm and assigning them to different colors for visualization. All modules are summarized by the module feature (ME) gene. The module feature gene is the most important component of each module and is calculated as a synthetic gene representing the expression profile of all genes in a given module.

### Identification of Differentially Expressed Genes Related to Ferroptosis

In order to determine the DEGs between sepsis and healthy control, we applied the empirical Bayesian method of the limma R package. The criteria for DEGs are set to |logFC| >0.5, and the P-value is <0.05.

The sequencing data in the sepsis cell model were divided into the control group and the LPS2h group; the control group was compared against the LPS9h group, both using the same standard (|logFC| >0.5 and P < 0.05). DEGs are converted into homologous human genes through the biomaRt package.

The DEGs between the most relevant modules of WGCNA and GSE65682, the DEGs between the control cell group and LPS2h, and the DEGs between the control cell group and LPS9h were used to obtain the ferroptosis-related genes. The ggplot2 package (17) was used to draw the volcano and heat map of DEGs as well as the Venn diagram to show the intersection genes. The intersected genes were then grouped by high and low expression level, and the clinical data in GSE65682 were used for survival analysis. The molecules that were meaningful for survival outcome were explored further.

### Single Gene Expression and Organ Distribution

The LPIN1 gene was selected based on the above analysis for further research, and the expression levels of LPIN1 were compared in blood samples of sepsis or the normal control and in the sequencing data of the sepsis cell model. The pROC package was used to build an receiver operating characteristic curve (ROC) prediction model to detect the different expressions. Using GTEx data, the expression level of LPIN1 was extracted in normal tissue and organs.

### Differentially Expressed Gene Screening and Functional Enrichment Analysis

According to the expression levels of LPIN1, the intersected DEGs were divided into a high expression group and a low expression group. The DEGs of the GSE65682 data set were screened by the Limma package, and the volcano map of DEGs was drawn using the ggplot2 package to show the DEGs, which satisfy P < 0.05 and |log2FC| >0.2.

Gene Ontology (GO) analysis is a common method for large-scale functional enrichment, including biological processes (BPs), molecular functions (MFs), and cellular components (CCs). Kyoto Encyclopedia of Genes and Genomes (KEGG) is a widely used database that stores information about genomes, biological pathways, diseases, and drugs. Using the GOplot package to perform GO and KEGG enrichment analyses, the cutoff value of false discovery rate (FDR) <0.05 was considered to be statistically significant and to visualize GO and KEGG results. We further used KEGG results to visualize the inflammation-related KEGG enrichment with the Pathview package (18).

### Gene Set Enrichment Analysis and Gene Set Variation Analysis

In order to study the differences in biological processes between different groups based on the gene expression profile of patients with sepsis, we used Gene Set Enrichment Analysis (GSEA) for gene set enrichment analysis. GSEA is a calculation method that analyzes whether a specific gene set has a statistical difference between two biological states. It is usually used to estimate the changes in pathways and biological process in the expression data set. The h.all.v7.4.entrez.gmt gene set was downloaded from the MSigDB database, the fgsea package (19) was used for GSEA, and adj.P.val <0.05 was considered statistically significant.

Gene Set Variation Analysis (GSVA) (20) is a non-parametric, unsupervised algorithm. GSVA can calculate the enrichment score of a specific gene set in each sample. Based on the c2.cp.v7.4.symbols.gmt data set, we used the expression profile data of sepsis patients to analyze the corresponding biological characteristics and observe the changes of related pathways to visualize with heat maps and volcano maps.

### Construction of Protein–Protein Interaction Network

In this study, we used the STRING database (http://string-db.org, version 11.09) online tool (21), which aims to predict protein–protein interaction (PPI) to construct a PPI network of selected genes with a score greater than 0.7 to construct a network model visualized by Cytoscape (v3.7.2) (22). Next, we used CytoHubba (23) to screen the top 20 key genes by score.

Then, we used the NetworkAnalyst online tool (24) to construct a gene regulation network of the hub gene–transcription factor (gene-TF) interaction network. In order to construct the gene-TF network, we used the TF and gene target data obtained from the ENCODE ChIP-seq data (25). According to the BETA Minus algorithm, only the peak intensity signal <500 and the predicted regulation potential score <1 are included. The NetworkAnalyst is used to construct a protein–chemical interaction network. The protein–chemical interaction comes from the Comparative Toxicogenomics Database (26), which contains the interactions between chemical substances and genes in the literature.

### Evaluation and Analysis of Immune Cell Infiltration and Correlation Analysis Between Immune Cells

CIBERSORT (27) is based on the principle of linear support vector regression to deconvolve the transcriptome expression matrix and estimate the composition and abundance of immune composition in the mixed cells. We uploaded the gene expression matrix data to CIBERSORT, filtered out samples with P < 0.05, and developed the immune cell infiltration matrix. The ggplot2 package (17) was used to draw bar graphs to show the distribution of 22 immune cell infiltrations in each sample; the corrplot package (28) was applied to draw related heat maps to visualize the correlation of 22 immune cell infiltrations. The ggplot2 package was further used to visualize the correlation between LPIN1 and immune cells as well as the differences in immune cells in different LPIN1 expression groups.

### Clinical Correlation Analysis

In order to study the clinical factors and the value of LPIN1 for clinical prognosis, the survival package and survminer package were used to perform single-factor and multifactor Cox regression analysis for LPIN1 on the clinical factors of GSE65682, to construct a multifactor Cox regression model, and to visualize with the forest plot. A nomogram chart was established based on the multivariate Cox model to predict the 28-day survival period of patients with sepsis. Finally, the calibration curve was used to evaluate the accuracy and resolution of the nomogram.

### Animal and Cell Culture

Healthy 8-week-old male rats were purchased from the Animal Laboratory of Nanchang University. The rats were housed in sterile cages with a humidity of 45%–55% and a light/dark cycle for 12 h, and they were bred adaptively for 1 week before the animal experiment. All animal experiments were performed in accordance with the “Guidelines for the Care and Use of Experimental Animals” (approval number: 81960346) approved by the Ethics Committee of Nanchang University.

A rat sepsis model [cecum ligation and puncture (CLP)] was established, and venous blood samples were obtained. The rats were anesthetized with sodium pentobarbital (50 mg/kg, intraperitoneal injection), and a 1.5–2-cm incision was made along the midline of the abdomen to expose the cecum. After the mesentery was stripped, except for the sham operation group, the cecum was ligated with a fourth thread at the end of the cecum. Except for mice in the sham operation group, a 21-gauge sterile needle was used to puncture the cecum at 1 cm of the distal end of the ligation, and the wound was sutured. For fluid resuscitation, mice received 1 ml of pre-warmed saline (37°C). For analgesia treatment, mice were injected with buprenorphine (0.05 mg/kg, subcutaneously) every 6 h for 2 consecutive days.

Rat lung macrophage NR8383 cells and rat lung epithelial (RLE) type II RLE-6TN cells were seeded in a 6-well plate containing 2 ml medium per well at a density of 10^6^ cells. When the cells grew to 50%, 1 μg/ml of LPS was used to stimulate cells. LPS-treated cells and untreated NR8383 and RLE-6TN cells were collected after 2 h and 9 h, respectively, to establish a sepsis cell model and obtain cell specimens.

### RNA Extraction and Real-Time Quantitative PCR

Total RNA was extracted using TRIzol reagent (Takara, Dalian, China). Prime-Script RTase (Takara) was used for reverse transcription. With the help of the premix Ex-Taq (Takara), the gene expression level was determined by qPCR and normalized to the glyceraldehyde-3-phosphate dehydrogenase (GAPDH). We used the 2-^ΔCT^ method to calculate the expression level.

### Statistical Analysis

All data processing and analyses were done through R software (version 4.1.0). For the comparison of two groups of continuous variables, the statistical significance of normally distributed variables was estimated by independent Student’s t-test, and the differences between non-normally distributed variables were analyzed by Mann–Whitney U test (Wilcoxon rank sum test). Chi-square test or Fisher’s exact test was used to compare and analyze the statistical significance between the two groups of categorical variables. All statistical P-values were two-tailed, and P < 0.05 was considered statistically significant.

## Results

### Weighted Gene Co-Expression Network Analysis

**Figure 1:**
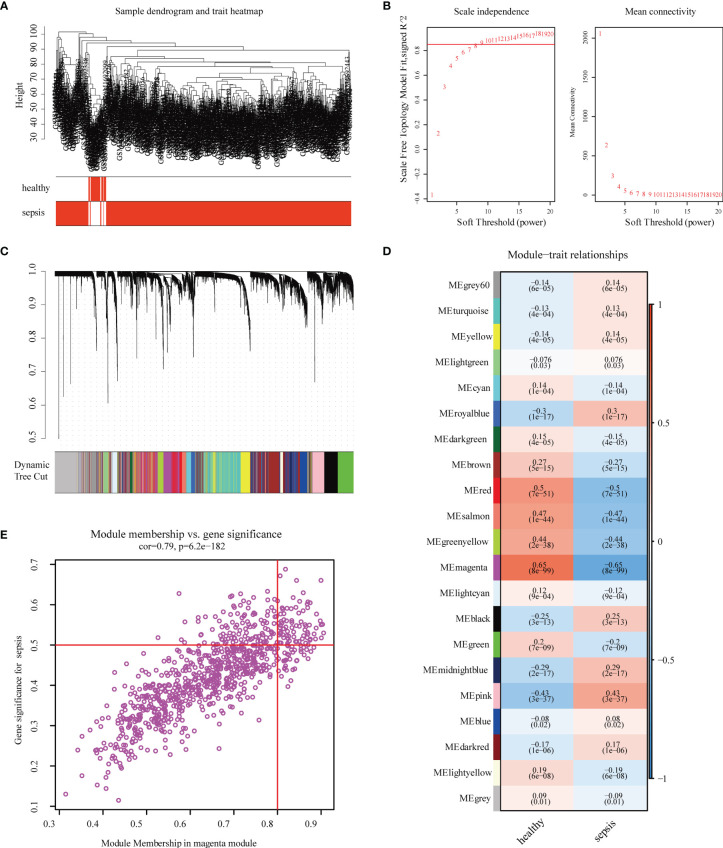
Construction and module analysis of weighted gene co-expression network analysis (WGCNA). **(A)** Sample clustering dendrogram based on Euclidean distance. **(B)** Network topology analysis under various soft-threshold powers. Left: The x-axis represents the soft-threshold power. The y-axis represents the fit index of the scale-free topology model. Right: The x-axis represents the soft-threshold power. The y-axis reflects the average connectivity (degree). **(C)** Clustering dendrogram of genes with different similarities based on topological overlap and the assigned module color. **(D)** Module–trait association. Each row corresponds to a module, and each column corresponds to a feature. Each cell contains the corresponding correlation and P-value. This table is color-coded according to the relevance of the color legend. **(E)** The relevance of members in the magenta module and sepsis.

According to the clinical characteristics of sepsis, the healthy control group and the sepsis group were analyzed by cluster analysis of their gene expression profiles (**Figure 1A**). To ensure that the network is scale-free, we chose a soft threshold of β = 9 (**Figure 1B**). Next, we transformed the expression matrix into an adjacency matrix and then transformed it into a topological matrix. The average linkage hierarchical clustering method was used to cluster genes. We also set the minimum number of genes in each gene network module to 50 according to the standard of hybrid dynamic shearing tree, which is used to determine the gene modules, and the characteristic gene value of each module was calculated. The parameters were as follows: height (MEDissThres) = 0.10, depth split (deepSplit) = 2, minimum module (minModuleSize) size = 50; and a total of 21 modules were obtained (**Figure 1C**). The module with the highest correlation with sepsis was magenta, r = -0.65, P = 8e -99 (**Figure 1D**). The significance of the genes in the magenta module with the sepsis genes was cor = 0.79, P = 6.2e-182 (**Figure 1E**).

### Differential Expression Analysis and Determination of the Target LPIN1

**Figure 2:**
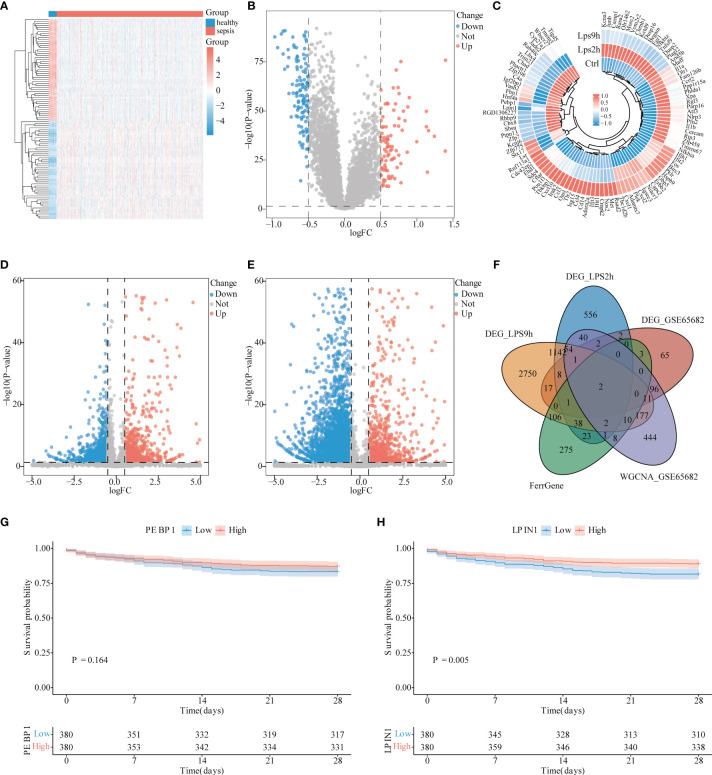
Differential analysis of sepsis. **(A)** Heat map of differential analysis between sepsis and control group. Light blue is the control group, red is the sepsis group, blue square represents low expression, and red square represents high expression. **(B)** Volcano graph of the control group and sepsis group in differential analysis. The blue dots represent low expression, while the red dots represent high expression. **(C)** Heat map of the expressional changes in NR8383 cells before and after lipopolysaccharide (LPS) 2 h, 9 h (Ctrl, LPS2h, LPS9h). Blue dots represent low expression, and red dots represent high expression. **(D)** Volcano diagram for difference analysis of NR8383 cells before and 2 h after LPS addition. Blue dots represent low expression, and red dots represent high expression. **(E)** Volcano diagram for difference analysis of NR8383 cells before and 9 h after LPS addition. The blue point of the volcano chart represents low expression, and the red point represents high expression. **(F)** Venn figure for intersected genes in DEG_LPS2h, DEG_LPS0h, DEG_GSE65682, Ferr gene, and WGCNA_GSE65682. **(G)** PEBP1 survival analysis in patients with sepsis. The blue line represents low expression group, and the red line represents high expression group, P = 0.164. **(H)** LPIN1 survival analysis in patients with sepsis. The blue line represents the low expression group, and the red line represents the high expression group, P = 0.005.

After dividing GSE65682 into the normal control group and the sepsis group, the difference analysis was performed with the Limma package, setting the P-value <0.05 and |log2FC| >0.5, and 208 DEGs were obtained. Among which, 120 were low-expressed genes and 88 genes are highly expressed. The results are visualized with a heat map (**Figure 2A**) and a volcano map (**Figure 2B**).

After obtaining the cell sequencing data, we divided the data into the control group, the group 2 h after LPS stimulation (LPS2h), and the group 9 h after LPS stimulation (LPS9h). Using the Limma package for difference analysis, and setting P-value <0.05 and |log2FC| >0.5, 2,167 DEGs in the Ctrl-LPS2h group were obtained, of which 1,263 were low-expressed genes, 904 were high-expressed genes (**Figures 2C, D** **)**, and 4,911 were DEGs in the Ctrl-LPS9h group, of which 3,902 were low-expressed genes and 1,017 high-expressed genes were expressed (**Figures 2D, E** **)**.

We intersected 208 DEGs in GSE65682, 2,167 DEGs in LPS2h, and 4,911 DEGs in LPS2h along with 848 magenta modular genes and 470 ferroptosis-related genes to obtain PEBP1 and LPIN1 genes (**Figure 2F**). However, LPIN1 was not among DEGs with adj. P-value <0.05. Furthermore, we used the survival data of sepsis patients in GSE65682 to perform the survival analysis according to the expression levels of PEBP1 and LPIN1. We found that the P-value of PEBP1 was 0.164 (**Figure 2G**), and the P-value of LPIN1 was 0.005 (**Figure 2H**). The LPIN1 was selected for subsequent analysis.

### Low Expression of LPIN1 in Sepsis

**Figure 3:**
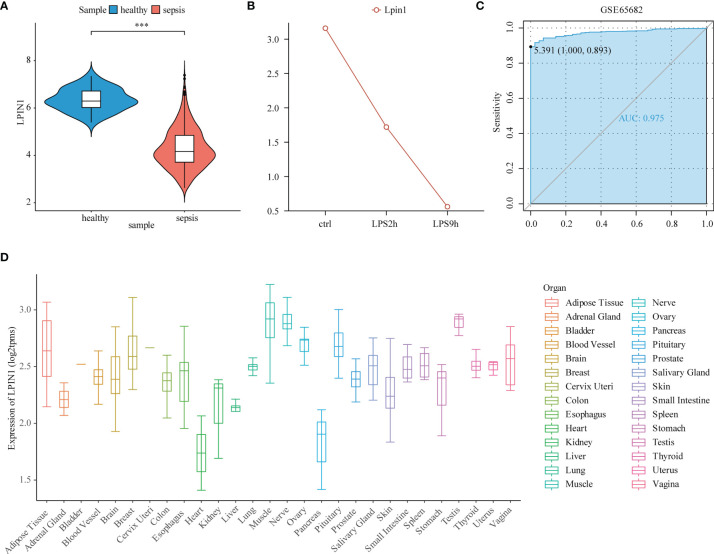
The expression pattern of LPIN1 in sepsis. **(A)** Reduced LPIN1 expression level in sepsis patients compared to normal donors from the GSE65682 dataset. **(B)** LPIN1 expression level in NR8383 cells before lipopolysaccharide (LPS) stimulation, 2 h after LPS stimulation, and 9 h after LPS stimulation. **(C)** The ROC of LPIN1 expression in GSE65682 to distinguish the normal and sepsis group with an AUC of 0.975. **(D)** LPIN1 expression profiling in the whole-body tissues and organs. ***P<0.001.

In the GSE65682 data set, the LPIN1 expression levels of the normal donor group and the sepsis group were extracted, and the Wilcox test detected the different expressions between the two groups. The expression of LPIN1 in the sepsis group was lower than that of the normal group (**Figure 3A**). In the sequencing data of the sepsis cell model, LPIN1 also showed a downward trend with the passage of time after LPS stimulation (**Figure 3B**). In the GSE65682 data, the area under the ROC curve (AUC) of LPIN1 to significantly distinguish between the normal group and the sepsis group was 0.975 (**Figure 3C**). The expression distribution of LPIN1 in the whole body was also different, with the highest expression in muscle tissue, followed by testis and nerves, and medium expression in blood and lung tissues (**Figure 3D**).

### Difference Analysis and Enrichment Analysis of LPIN1 Grouping

**Figure 4:**
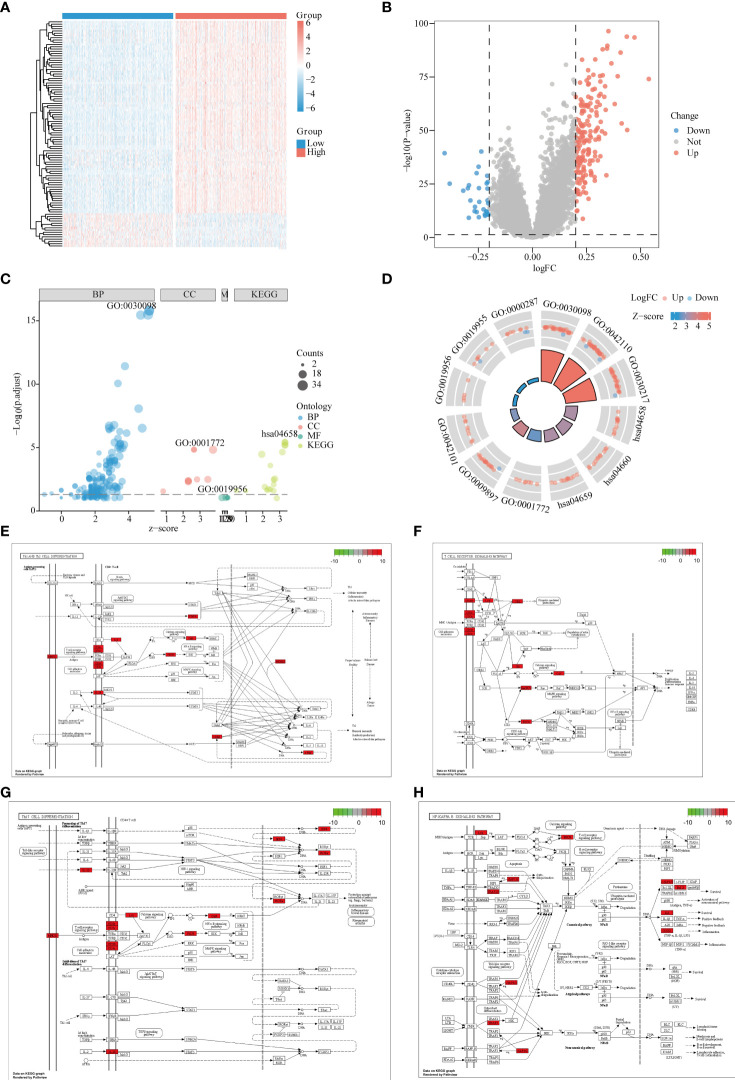
Differential and enrichment analysis of LPIN1 grouping. **(A)** In GSE65682, the differential analysis heat map of LPIN1 high and low expression groups. Blue is the LPIN1 low expression group, and red is the LPIN1 high expression group. **(B)** In GSE65682, differential analysis volcano graph for LPIN1 expression. Blue dots represent low expression, and red dots represent high expression. **(C)** Differentially expressed genes (DEGs) in Gene Ontology (GO) and Kyoto Encyclopedia of Genes and Genomes (KEGG) analysis. Light blue represents BP functional enrichment, light red represents CC functional enrichment, light green represents MF functional enrichment, oyster represents KEGG pathway enrichment, and the size of the dot represents the number of gene counts. **(D)** Circle map for DEGs in GO and KEGG analysis. Each column in the inner circle corresponds to the relative size of P.adj. of an entry; the higher the column, the smaller P.adj. The column color corresponds to the z-score value. The outer circle is the molecule in the entry, and the different heights represent the corresponding logFC value. Molecules with positive logFC are marked as Up, light red, and molecules with negative logFC are marked as Down, light blue. **(E–H)** DEGs in Th1 and Th2 cell differentiation pathway. **(E)** T-cell receptor signaling pathway. **(F)** Th17 cell differentiation pathway. **(G)** NF-κB signaling pathway. **(H)** Red indicates high expression in the pathway, and green indicates low expression in the pathway.

GSE65682 was divided into a high-expression group and a low-expression group according to the median value of LPIN1, with P < 0.05 and |log2FC| >0.2; 29 low-expressed genes and 176 high-expressed genes were obtained. The heat map only showed the top 100 DEGs in |logFC| order (**Figures 4A, B** **)**.

In order to further study the functional effects of different LPIN1 expressions, we first analyzed the 205 DEGs of GSE65682 by GO and KEGG. The top two groups functional effects of BP, CC, MF, and KEGG were lymphocyte differentiation, T-cell activation, T-cell differentiation, immunological synapse, external side of plasma membrane, T-cell receptor (TCR) complex, chemokine binding, cytokine binding, magnesium ion binding, Th1 and Th2 cell differentiation, TCR signaling pathway, and Th17 cell differentiation (**Figures 4C, D** and **Table 1**). Functional enrichment could clearly see that the expression of LPIN1 was closely related to T cells and immunity.

**Table 1:** GO and KEGG enrichment analysis for DEGs.

Ontology	ID	Description	Count	P-value
BP	GO:0030098	Lymphocyte differentiation	31	5.08e-20
BP	GO:0042110	T-cell activation	34	2.08e-19
BP	GO:0030217	T-cell differentiation	26	3.43e-19
CC	GO:0001772	Immunological synapse	7	6.47e-08
CC	GO:0009897	External side of plasma membrane	18	1.14e-07
CC	GO:0042101	T-cell receptor complex	8	4.66e-05
MF	GO:0019956	Chemokine binding	4	4.12e-04
MF	GO:0019955	Cytokine binding	7	5.61e-04
MF	GO:0000287	Magnesium ion binding	9	6.29e-04
KEGG	hsa04658	Th1 and Th2 cell differentiation	11	1.74e-08
KEGG	hsa04660	T-cell receptor signaling pathway	11	6.36e-08
KEGG	hsa04659	Th17 cell differentiation	11	8.56e-08

DEG, differentially expressed gene; GO, Gene Ontology; KEGG, Kyoto Encyclopedia of Genes and Genomes.

Using KEGG analysis data, the Pathview package showed that the Th1 and Th2 cell differentiation pathway (**Figure 4E**), TCR signaling pathway (**Figure 4F**), Th17 cell differentiation pathway (**Figure 4G**), and nuclear factor (NF)-κB signaling pathway (**Figure 4H**) all have significant gene expressions.

### Gene Set Enrichment Analysis and Gene Set Variation Analysis Enrichment Analysis

**Figure 5:**
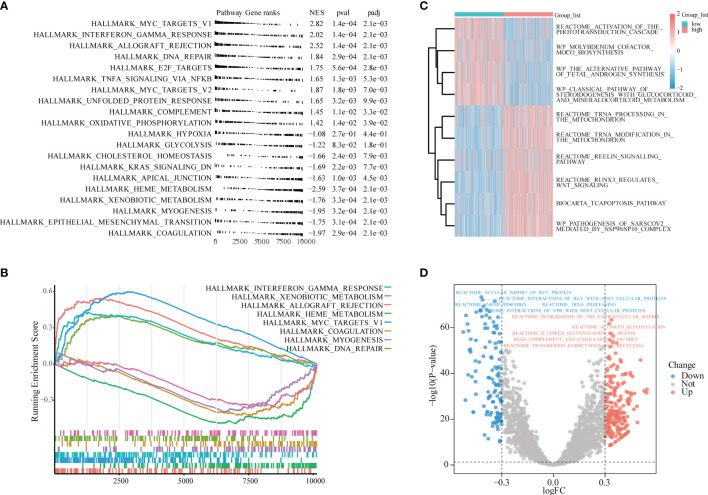
Gene Set Enrichment Analysis (GSEA), Gene Set Variation Analysis (GSVA) enrichment analysis. **(A)** GSEA. **(B)** The most significant eight gene sets shown in GSEA. **(C)** LPIN1 high and low expression groups for GSVA enrichment analysis. The heat map shows the 10 most significant gene sets; light blue is the LPIN1 low expression group, light red is the LPIN1 high expression group, the blue square represents low enrichment, and the red square represents high enrichment. **(D)** Volcano map for differential GSVA enrichment analysis of LPIN1 expression high and low groups. Blue dots represent low enrichment, and red dots represent high enrichment.

To avoid the one-sidedness caused by only using the intersection gene enrichment, we also performed GSEA on all genes of sepsis patients in GSE65682. The reference gene set was h.all.v7.4.symbols.gmt, and the one with the smallest P-value was selected. Eight related datasets displayed tumor necrosis factor (TNF)α signaling *via* NF-κB, HallMark_Hypoxia, HallMark_cholesterol homeostasis, HallMark_DNA repair, HallMark_myogenesis, HallMark_interferon-γ response, HallMark_apical junction, and HallMark_complement (**Figures 5A, B** and **Table 2**). These pathways were also mostly related to inflammation, oxidative stress, immunity, glycolysis, and other similar effects.

**Table 2:** GSEA.

Description	Set Size	Enrichment Score	NES	P-value
HALLMARK_TNFA_SIGNALING_VIA_NFKB	151	0.3591	1.6440	0.0015
HALLMARK_HYPOXIA	138	-0.2142	-1.0824	0.2630
HALLMARK_CHOLESTEROL_HOMEOSTASIS	57	-0.3896	-1.6695	0.0047
HALLMARK_DNA_REPAIR	132	0.4076	1.8336	0.0001
HALLMARK_MYOGENESIS	114	-0.3978	-1.9461	0.0003
HALLMARK_INTERFERON_GAMMA_RESPONSE	176	0.4308	2.0132	0.0001
HALLMARK_APICAL_JUNCTION	127	-0.3278	-1.6335	0.0013
HALLMARK_COMPLEMENT	154	0.3157	1.4484	0.0144
HALLMARK_UNFOLDED_PROTEIN_RESPONSE	93	0.3875	1.6529	0.0024
HALLMARK_E2F_TARGETS	154	0.3794	1.7407	0.0006
HALLMARK_MYC_TARGETS_V1	182	0.6001	2.8179	0.0001
HALLMARK_MYC_TARGETS_V2	45	0.5055	1.8759	0.0005
HALLMARK_EPITHELIAL_MESENCHYMAL_TRANSITION	104	-0.3649	-1.7606	0.0003
HALLMARK_XENOBIOTIC_METABOLISM	132	-0.3502	-1.7576	0.0003
HALLMARK_OXIDATIVE_PHOSPHORYLATION	177	0.3033	1.4183	0.0162
HALLMARK_GLYCOLYSIS	145	-0.2383	-1.2120	0.0941
HALLMARK_HEME_METABOLISM	176	-0.4939	-2.5907	0.0004
HALLMARK_COAGULATION	83	-0.4265	-1.9686	0.0003
HALLMARK_ALLOGRAFT_REJECTION	157	0.5469	2.5160	0.0001
HALLMARK_KRAS_SIGNALING_DN	62	-0.3902	-1.7003	0.0031

GSEA, Gene Set Enrichment Analysis.

The GSVA reference gene set selected c2.cp.v7.4.symbols.gmt. There were significant differences in the gene set in LPIN1, and the top 10 subtypes were as follows: activation of the phototransduction cascade, molybdenum cofactor moco biosynthesis, the alternative pathway of fetal androgen synthesis, the tcapoptosis pathway, and the reelin signaling pathway.

Runx3 regulates WNT signaling, the classical pathway of steroidogenesis with glucocorticoid and mineralocorticoid metabolism, pathogenesis of sarscov2_mediated by the NSP9NSP10 complex, tRNA processing in the mitochondrion, and tRNA modification in the mitochondrion.

These different gene sets are related to the WNT pathway, mitochondrial metabolism, hormone action, and other pathways (**Figures 5C, D** and **Table 3**).

**Table 3:** GSVA Enrichment Analysis.

Pathway Name	logFC	AveExpr	t	P-value	B
REACTOME_ACTIVATION_OF_THE_PHOTOTRANSDUCTION_CASCADE	0.5439	-0.0081	12.7125	9.95E-34	65.6634
WP_MOLYBDENUM_COFACTOR_MOCO_BIOSYNTHESIS	0.5417	-0.0049	12.3863	3.03E-32	62.2763
WP_THE_ALTERNATIVE_PATHWAY_OF_FETAL_ANDROGEN_SYNTHESIS	0.5278	-0.0061	12.5611	4.89E-33	64.0841
BIOCARTA_TCAPOPTOSIS_PATHWAY	-0.5241	0.0024	-17.6534	9.04E-59	122.9248
REACTOME_REELIN_SIGNALLING_PATHWAY	-0.5116	-0.0077	-11.6585	5.00E-29	54.9334
REACTOME_RUNX3_REGULATES_WNT_SIGNALING	-0.5083	-0.0039	-18.9565	5.38E-66	139.4623
WP_CLASSICAL_PATHWAY_OF_STEROIDOGENESIS_WITH_GLUCOCORTICOID_AND_MINERALOCORTICOID_METABOLISM	0.5072	0.0191	15.3456	1.55E-46	94.9368
WP_PATHOGENESIS_OF_SARSCOV2_MEDIATED_BY_NSP9NSP10_COMPLEX	-0.4993	0.0196	-18.2966	2.60E-62	131.0303
REACTOME_TRNA_PROCESSING_IN_THE_MITOCHONDRION	-0.4952	0.0014	-14.0497	4.65E-40	80.1250
REACTOME_TRNA_MODIFICATION_IN_THE_MITOCHONDRION	-0.4930	-0.0031	-17.7494	2.70E-59	124.1264

GSVA, Gene Set Variation Analysis.

### Protein–Protein Interaction Network and Related Functional Network Construction

**Figure 6:**
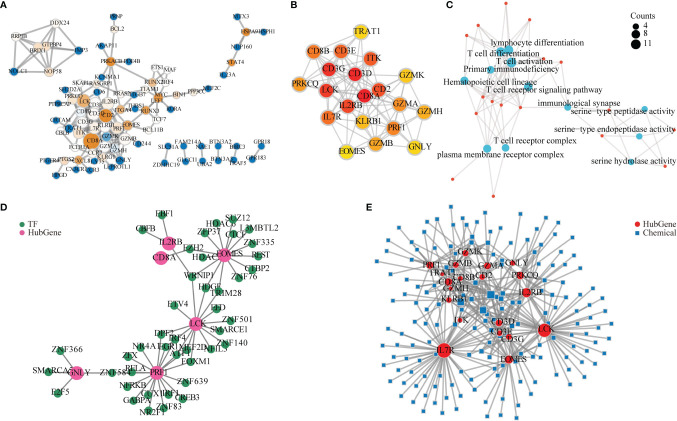
Protein–protein interaction (PPI) network and related functional network analysis. **(A)** The 88 differentially expressed genes (DEGs) in PPI network constructed by the STRING database. The larger value of betweenness centrality is brown, while the smaller is blue. A larger node degree value is indicated by a larger diameter of the point. **(B)** CytoHubba calculated 20 hub genes. A darker color indicates a higher score. **(C)** Gene Ontology (GO) and Kyoto Encyclopedia of Genes and Genomes (KEGG) analysis for 20 hub genes. A bigger circle indicates a higher score. **(D)** Hub gene–transcriptional factor (TF) network. Light red represents a hub gene, and green represents TF. **(E)** mRNA–chemical interactive network. The red circle represents mRNA, and the green square represents small molecule compounds. A larger degree is indicated by a larger diameter of the dot.

In order to further explore its mechanism, we used Cytoscape to construct a PPI network based on the STRING database. We concluded that 88 genes had 189 connections (**Figure 6A**), and the CytoHubba plug was used to screen the top 20 key genes by score. There were 95 connections in 20 hub genes (**Figure 6B**).

To explore the function of these hub genes, GO and KEGG analysis were applied. The results revealed that their function mainly involves immune cell activation and differentiation, immunological synapse, and primary immunodeficiency (**Figure 6C**).

To explore the function of hub genes in TFs, we used the NetworkAnalyst online tool to predict the TFs of 20 hub genes and to build an mRNA–TF network. Among the hub genes, six hub genes had TFs to form a network. The network had 49 nodes and 55 connections (**Figure 6D**).

As hub genes may carry out the dominant role, we also continued to explore the interaction between hub genes and small molecule compounds and provided references for possible therapeutic drugs. We used the NetworkAnalyst online tool to predict compounds from 20 hub genes and construct an mRNA–chemical network. Among them, 20 hub genes and 168 small molecule compounds that form the network were identified, which included 188 nodes and 358 connections in the network (**Figure 6E**).

### Evaluation and Analysis of Immune Cell Infiltration

**Figure 7:**
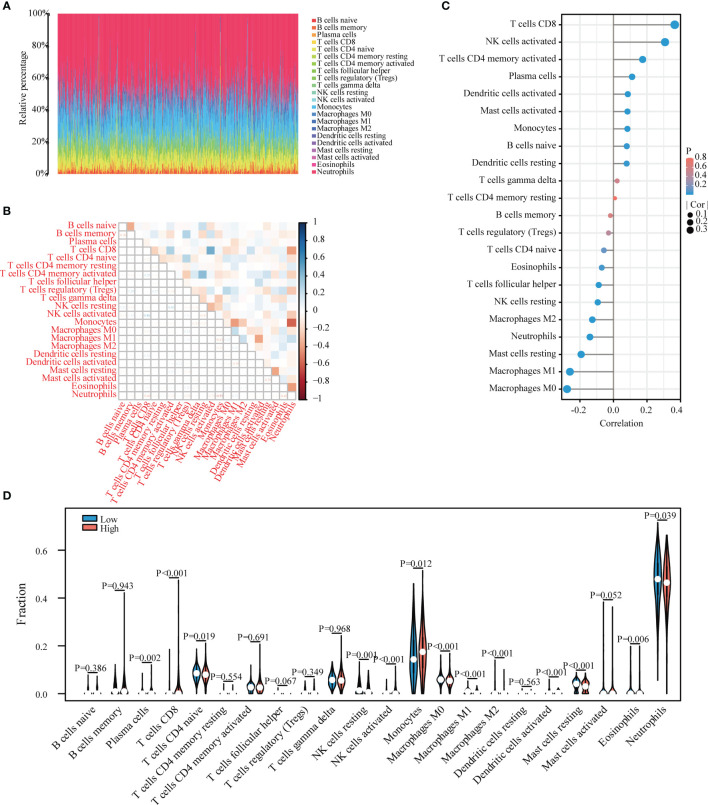
Evaluation of immune cell infiltration and correlational analysis. **(A)** Barplot shows the proportion of 22 types of immune cells in sepsis samples. The column of the figure is listed as a sample. **(B)** The correlation heat map of 22 kinds of infiltrated immune cells. Blue means positive correlation, and red means negative correlation. A darker color indicates a stronger correlation. **(C)** The correlation between LPIN1 and immune cells is lollipop graph. Blue represents a smaller P-value, and red represents a higher P-value. A larger dot indicates a higher correlation. The left of the figure is a negative correlation, and the right part is a positive correlation. **(D)** Twenty-two kinds of immune cells are expressed at different levels of LPIN1 (low vs. high) as violin chart. Blue is the low expression group, and red is the high expression group.

In order to further confirm the correlation between LPIN1 expression and immune cells, the CIBERSORT algorithm was used to analyze the proportion of immune cell infiltration, and a map of 22 immune cells in sepsis samples (**Figure 7A**) and their correlation with 22 immune cells (**Figure 7B**) were constructed. The correlation analysis between LPIN1 and immune cells showed that a total of 16 kinds of immune cells had significant correlations with LPIN1. M0 macrophages, M1 macrophages, M2 macrophages, mast cells resting, neutrophils, resting natural killer (NK) cells, and T follicular helper cells were negatively correlated with LPIN1 expression. There was a positive correlation between the expression of resting dendritic cells, naive B cells, monocytes, mast cells activated, activated dendritic cells, plasma cells, T cells CD4 memory activated, activated NK cells, and T cells CD8 and LPIN1 expression (**Figure 7C**). We further analyzed the correlation of immune cell scores according to the expression level of LPIN1 and found that different immune cells have different performances in the two groups. Among them, activated dendritic cells, eosinophils, M0 macrophages, M1 macrophages, M2 macrophages, mast cells resting, monocytes, neutrophils, activated NK cells, resting NK cells, plasma cells, naive T cells CD4, and T cells CD8 are quite different (**Figure 7D**). These results further support that the level of LPIN1 might affect the immune activity of immune cells.

### Clinical Correlation Analysis

**Figure 8:**
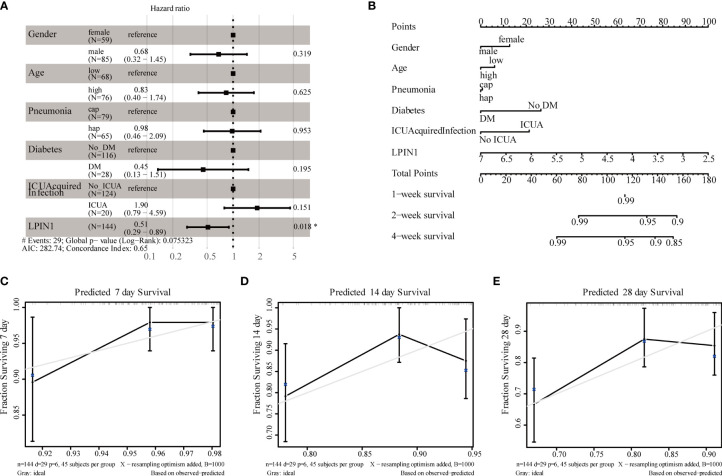
Clinical correlational analysis. **(A)** Forest plot for multivariate Cox regression analysis. **(B)** Nomogram plot for multivariate Cox regression analysis. **(C–E)** 7-day, 14-day, 28-day survival rate calibration curve.

There was a significant correlation between LPIN1 and the survival of patients with sepsis. We used the clinical data in GSE65682 (**Table 4**) to further analyze the impact of other clinical factors on survival. We included age, gender, whether the lung infection was community-acquired or hospital-acquired, presence of diabetes, and whether the infection was intensive care unit (ICU)-acquired using LPIN1 univariate and multivariate Cox regression analysis (**Table 5**); the multivariate Cox analysis showed that LPIN1 had a significant correlation with survival (**Figure 8A**). In order to explore the predictability of clinical factors on survival, a nomogram (**Figure 8B**) was established according to the multivariate results, especially on 7-day, 14-day, and 28-day survival rate (**Figures 8C–E**). The C-index value was 0.68, which indicated that this nomogram was predictable, also reflected by the calibration curve.

**Table 4:** Basic clinical characteristics in patients with high or low LPIN1.

	Overall	Low	High	*X^2^*	P
n	144	75	69		
Gender = female/male (%)	59/85 (41.0/59.0)	28/47 (37.3/62.7)	31/38 (44.9/55.1)	0.857	0.450
Age = low/high (%)	68/76 (47.2/52.8)	35/40 (46.7/53.3)	33/36 (47.8/52.2)	0.019	1.000
Pneumonia = cap/hap (%)	79/65 (54.9/45.1)	43/32 (57.3/42.7)	36/33 (52.2/47.8)	0.386	0.650
Diabetes = DM/No_DM (%)	28/116 (19.4/80.6)	16/59 (21.3/78.7)	12/57 (17.4/82.6)	0.356	0.699
ICUAcquiredInfection = ICUA/No_ICUA (%)	20/124 (13.9/86.1)	13/62 (17.3/82.7)	7/62 (10.1/89.9)	1.553	0.315
LPIN1 [mean (SD)]	4.23 (0.80)	3.63 (0.37)	4.88 (0.60)		<0.001

**Table 5:** Univariate and multivariate Cox analysis.

Variables	Univariate analysis				Multivariate analysis		
	HR	95% CI of HR	P-value		HR	95% CI of HR	P-value
Gender	0.83	0.39–1.71	0.6067		0.68	0.31–1.45	0.3191
Age	0.82	0.39–1.69	0.5883		0.83	0.39–1.74	0.6246
Pneumonia	0.86	0.41–1.8	0.6946		0.98	0.45–2.08	0.9534
Diabetes	0.46	0.13–1.5	0.1985		0.45	0.13–1.5	0.1950
ICU acquired infection	1.98	0.84–4.64	0.1151		1.90	0.79–4.58	0.1511
LPIN1	0.50	0.28–0.87	0.0160		0.51	0.29–0.89	0.0185

HR, hazard ratio; ICU, intensive care unit.

### LPIN1 and Related Pathway Variation

**Figure 9:**
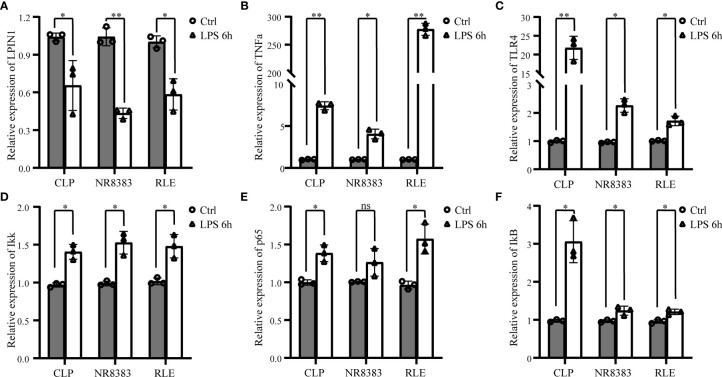
The verification of LPIN1 expression and related pathways in sepsis. Lipopolysaccharide (LPS) stimulation could decrease the mRNA level of Lpin1 in a sepsis rat model (CLP), NR8383 and RLE cell lines **(A)**, with the increased mRNA level in tumor necrosis factor (TNF)α, **(B)** Toll-like receptor (TLR)4, **(C)** IKK, **(D)** p65 subunit, **(E)** and IκB. **(F)** Comparison to the control group. *P < 0.05, **P < 0.01, ^ns^P < 0.05.; n = 3 in each group. CLP, cecum ligation and puncture; RLE, rat lung epithelial.

With the bioinformatic analysis, we found that sepsis was related to both inflammation and immune response. To confirm these findings, we also found that LPS stimulation could inhibit the expression of LPIN1 in a sepsis rat model. NR8383 and RLE cell lines (**Figure 9A**), with the facilitation of TNFα (**Figure 9B**), Toll-like receptor (TLR)4 (**Figure 9C**), IKK (**Figure 9D**), p65 subunit (**Figure 9E**), and IκB (**Figure 9F**), were compared to the control group.

## Discussion

This study applied WGCNA to identify the ferroptosis-related genes in sepsis and related modules. The bioinformatic analysis allowed us to identify that LPIN1 is a critical gene in sepsis. In addition, the *in vitro* and *in vivo* studies verified that LPS stimulation could inhibit the expression of LPIN1 with the facilitation of TNFα, TLR4, IKK, p65 subunit, and IκB (29).

To explore the function of LPIN1, we first identified its 20 hub genes with PPI network and CytoHubba. These genes were listed in **Figure 6B**. We also found that their functions were mostly involved in immune response with GO and KEGG analysis (**Figure 6D**). PEBP1 and LPIN1 were both intersected among GSE65682, LPS 2h, and LPS 9h datasets, which indicated that these two genes might have been involved in sepsis-induced ferroptosis. PEBP1, phosphatidylethanolamine-binding protein 1, is involved in ferroptosis in airway epithelial cells in asthma (30). It is also associated with immune cell inflammation (31). Consistently, a very recent study also suggested that increased expression of PEBP1 is related to survival status in lung adenocarcinoma patients (32). In our study, however, only LPIN1 is found to be correlated with survival status in sepsis. This might be due to the fact that PEBP1 is more closely associated with pulmonary diseases.

Next, we applied GO and KEGG analysis and found that LPIN1 is involved in lymphocyte differentiation, T-cell activation, T-cell differentiation, and immunological synapse in the GO analysis. The KEGG pathways were mainly involved in Th1, Th2, Th17 cell differentiation, TCR signaling, and the NF-κB pathway. In addition, the GSEA also suggested that TNF and NF-κB pathways were involved in sepsis, which indicated that inflammation was critically involved in sepsis.

Therefore, we further verified the mRNA level of LPIN1 after LPS stimulation and found its expression reduced in the sepsis rat model along with NR8383 and RLE cell lines, which were together with increased TNFα and TLR4 expression. Moreover, we found that NF-κB signaling markers such as Ikk, p65 subunit, and IκB were all increased after LPS stimulation. All of these findings were consistent with the pathway enrichment analysis.

LPIN1 was annotated to the most significantly enriched motifs for genes in the magenta module. The eigengene of the magenta module is most significantly associated with mortality, and thus, the master regulator LPIN1 is also a critical risk factor for mortality *via* immunomodulation (33, 34). Our findings were consistent with those of a previous study, finding that LPS stimulation could reduce the expression of lipin1 *via* activating TLR-4 and that TNF-α treatment also reduced the lipin1 expression in mice (35). However, an LPS-induced decrease in Lpin1 could be partly reversed in TNF-α and interleukin (IL)-1β knockout mice (35).

Our analysis has several implications for clinical practice. First, sepsis is shown to have a dysregulated immune response highlighted by the upregulation of the expression level of TLR4 (36–38). Immunological dysregulation is considered as a critical cause of sepsis (39, 40). However, most studies investigated individual genes at a high-throughput level involving DEGs, followed by enrichment pathway analysis (4, 41, 42).

However, there were some limitations in this study. First, the severity of sepsis was not classified in our study. In a future study, we must enroll clinical patients to evaluate whether LPIN1 could become a biomarker for sepsis. Second, the ferroptosis-related regulators were predicted by a bioinformatic study based on GEO and our home-costumed RNA sequencing (RNA-seq) data, which might have a high false-positive rate. The *in vivo* function of these genes should be verified in experimental studies. Third, there are other causes of sepsis that were not explored in the current study. For example, peripheral infection is also a critical cause of sepsis; however, both the GEO data or our experimental model did not contain this kind of information, which needs to be supplemented in future analyses.

In conclusion, the present study identified that LPIN1 is statistically associated with mortality in sepsis. Related pathways of LPIN1 include inflammation and immune response, which indicates that LPIN1 might be a potential therapeutic target for sepsis.

## Data Availability Statement

The datasets presented in this study can be found in online repositories: https://www.jianguoyun.com/p/DXp3a8cQzu6GChi4iJ0E. The names of the repository/repositories and accession number(s) can be found in the article/supplementary material.

## Ethics Statement

The animal study was reviewed and approved by the ethics committee of Nanchang University.

## Author Contributions

WD and PZ conceived the idea. WD designed the study. DL, QX, FL, QS, and NZ collected the data and performed the experiments. PZ drafted the article. WD and PZ reviewed and corrected the article. KQ provided supervision, project administration, funding acquisition, and paper finalization. All authors contributed to the article and approved the submitted version.

## Funding

This work was supported by grants from the National Natural Science Foundation of China (No. 81871548, No. 82160363). This work was also supported by The Featured Clinical Discipline Project of Shanghai Pudong (PWYst2018-01).

## Conflict of Interest

The authors declare that the research was conducted in the absence of any commercial or financial relationships that could be construed as a potential conflict of interest.

## Publisher’s Note

All claims expressed in this article are solely those of the authors and do not necessarily represent those of their affiliated organizations, or those of the publisher, the editors and the reviewers. Any product that may be evaluated in this article, or claim that may be made by its manufacturer, is not guaranteed or endorsed by the publisher.
